# Sowing an Idea to Harvest a Better Future

**DOI:** 10.21470/1678-9741-2024-0166

**Published:** 2025-03-11

**Authors:** Francisco Candido Monteiro Cajueiro

**Affiliations:** 1 Division of Pediatric Cardiovascular Surgery - Universidade de Pernambuco - PROCAPE/UPE, Recife, Pernambuco, Brazil. E-mail: emaildefrancisco@gmail.com; 2 Real Hospital Português de Beneficência em Pernambuco - RHP, Recife, Pernambuco, Brazil

Dear Editor,

I would like to express my congratulations to the Brazilian Journal of Cardiovascular Surgery for publishing the special article "Challenges of Congenital Heart Surgery in Brazil: It is Time to Designate Pediatric Congenital Heart Surgery Subspecialty"^[1]^ and to offer my perspective as a young cardiovascular surgeon with training in pediatric cardiovascular surgery in Brazil.

Providing care for children with congenital heart diseases truly represents a test for the entire healthcare system. As outlined by the authors, the array of challenges involved in addressing this demand is diverse and particularly exacerbated in our country.

Pediatric cardiovascular surgery is a highly multidisciplinary field that demands qualification of all agents involved in the continuum of care. As observed in other countries, the unique aspects of caring for this population alone would justify the recognition of this surgical subspecialty, reaffirming the commitment of the Brazilian Society of Cardiovascular Surgery to children and adults with congenital heart disease.

For the current generation, exclusive dedication to pediatrics requires much more than just a vocation to help children. Achieving surgical maturity in this complex discipline requires a long journey of training, full dedication, the pursuit of technical proficiency, scientific knowledge, support from seniors, and a comprehensive alignment of institutional, local, regional, political, and economic backgrounds. It is frustrating, for many, to pursue this path in an area that simply does not exist and is not officially recognized.

Brazil is a breeding ground for excellent surgeons who have made significant contributions to the development of pediatric cardiovascular surgery worldwide. The recognition of the subspecialty will not only align Brazil with the global cause of congenital heart disease but also bring expectations for the consolidation of public policies, higher qualification of care, new educational opportunities and job positions.

The document brilliantly conceived by the Department of Pediatric Cardiovascular Surgery is more than just an article, it is a manifesto for the deserved affirmation of our history, capable of bringing hope to an entire generation of young surgeons.

*“If I have seen further, it is by standing on the shoulders of giants.”* (Sir Isaac Newton)
